# Impact of Residential Concentration of PM2.5 Analyzed as Time-Varying Covariate on the Survival Rate of Lung Cancer Patients: A 15-Year Hospital-Based Study in Upper Northern Thailand

**DOI:** 10.3390/ijerph19084521

**Published:** 2022-04-08

**Authors:** Nawapon Nakharutai, Patrinee Traisathit, Natthapat Thongsak, Titaporn Supasri, Pimwarat Srikummoon, Salinee Thumronglaohapun, Phonpat Hemwan, Imjai Chitapanarux

**Affiliations:** 1 Department of Statistics, Faculty of Science, Chiang Mai University, Chiang Mai 50200, Thailand; nawapon.nakharutai@cmu.ac.th (N.N.); patrinee.t@cmu.ac.th (P.T.); sittichai.thongsak@gmail.com (N.T.); pimwarat.n@cmu.ac.th (P.S.); salinee.t@cmu.ac.th (S.T.); 2 Data Science Research Center, Department of Statistics, Faculty of Science, Chiang Mai University, Chiang Mai 50200, Thailand; 3 Research Center in Bioresources for Agriculture, Industry and Medicine, Department of Statistics, Faculty of Science, Chiang Mai University, Chiang Mai 50200, Thailand; 4 Atmospheric Research Unit, National Astronomical Research Institute of Thailand, Chiang Mai 50180, Thailand; titasupasri@gmail.com; 5 Department of Geography, Faculty of Social Sciences, Chiang Mai University, Chiang Mai 50200, Thailand; phonpat.h@cmu.ac.th; 6 Northern Thai Research Group of Therapeutic Radiology and Oncology (NTRG-TRO), Divisions of Radiation Oncology, Faculty of Medicine, Chiang Mai University, Chiang Mai 50200, Thailand; 7 Chiang Mai Cancer Registry, Maharaj Nakorn Chiang Mai Hospital, Faculty of Medicine, Chiang Mai University, Chiang Mai 50200, Thailand

**Keywords:** lung cancer, mortality rate, PM2.5, PM10, survival rate

## Abstract

Air pollutants, especially particulate matter (PM) ≤ 2.5 µm (PM2.5) and PM ≤ 10 µm (PM10), are a major concern in upper northern Thailand. Data from a retrospective cohort comprising 9820 lung cancer patients diagnosed from 2003 to 2018 were obtained from the Chiang Mai Cancer Registry, and used to evaluate mortality and survival rates. Cox proportional hazard models were used to identify the association between the risk of death and risk factors including gender, age, cancer stage, smoking history, alcohol-use history, calendar year of enrollment, and time-updated PM2.5, PM10, NO_2_ and O_3_ concentrations. The mortality rate was 68.2 per 100 persons per year of follow-up. In a multivariate analysis, gender, age, cancer stage, calendar year of enrollment, and time-varying residential concentration of PM2.5 were independently associated with the risk of death. The lower the annually averaged PM2.5 and PM10 concentrations, the higher the survival probability of the patient. As PM2.5 and PM10 were factors associated with a higher risk of death, lung cancer patients who are inhabitant in the area should reduce their exposure to high concentrations of PM2.5 and PM10 to increase survival rates.

## 1. Introduction

Air pollution, mainly from outdoor pollution sources, kills more than 4.2 million people worldwide each year, and 9 out of 10 people live in places where the air quality is worse than the WHO guidelines [1]. As this is a long-term issue that is yet to be resolved, it is critical to comprehensively investigate the impact and provide solutions. In 2020, lung cancer accounted for 12.4% of new cancer cases and 16.3% of cancer-related deaths in Thailand, which was second only to liver cancer [2]. Meanwhile, a new strategy to enhance lung cancer treatment has been developed [3]. Lung cancer deaths in northern Thailand have increased steadily, at a rate much higher than in other regions of the country [4,5]. From 1997 to 2017, Lampang Cancer Hospital recorded an increase, from 1700 to 2400, in new lung cancer cases per year in the northern region, while the number of lung cancer-specific deaths rose from 1200 to 1800 per year [6]. 

Deaths of lung cancer patients are caused by various risk factors, the main one being smoking [7,8,9,10,11,12]. A large Norwegian study found that men who smoke had a 27-fold higher risk of death [12]. Other characteristics related to the mortality of lung cancer are sex (men have a higher risk than women) [7,12], age (a higher risk with increasing age) [7,10,11], body mass index (being overweight is riskier) [13,14], family history [8,10], and cancer stage [7,15]. Furthermore, external factors such as air pollution can have a significant effect on mortality; for example, PM (both PM ≤ 2.5 µm (PM2.5) [16,17,18,19,20,21,22,23] and PM ≤ 10 µm (PM10) [19,22,23,24,25,26,27]), NO_2_ [22,23] and O_3_ [28].

A PM crisis occurred in the first half of the year in major cities due to forest fires, the burning of agricultural waste (rice, corn, and sugarcane), cross-border pollution, and traffic and transport [29]. Accurate diagnosis of lung cancer (especially the staging) is crucial and must comply with international standards. Since excellent diagnostic tools are essential, and are only available in university-based hospitals, referring the many suspected lung cancer patients to them has created an inevitable burden on healthcare, equipment costs, and has inevitably complicated procedures. Moreover, socioeconomic status and economic conditions are also related confounding factors [30]. Chiang Mai province, surrounded by high mountains that block diffusion and redirect airflow, is an example of exacerbating PM accumulation along the foothills of mountains [31,32]. Therefore, this province has a problem with severe air pollution from PM [33]. Since the concentrations of PM, NO_2_ and O_3_ have been changing over time, they can be treated as time-varying covariates whose values can change over the duration of follow-up [34]. Cox proportional hazard models have been extensively used in the analysis of time-varying covariates, in order to investigate the association between events and variables over time [35]. Although Cox proportional hazard models have been applied in many survival-time studies, for instance [35,36,37,38], there is no study that has taken the concentration of PM, NO_2_ and O_3_ as the time-varying covariates when examining survival rates of lung cancer patients. To address this issue, our investigation will be the first study to use PM2.5, PM10, NO_2_, and O_3_ levels over the past 15 years as time-varying covariates in the Cox proportional hazard model to estimate the mortality rate, and to identify the risk factors associated with mortality among lung cancer patients. The data were collected during a 15-year hospital-based study in upper northern Thailand.

## 2. Methodology

### 2.1. The Study Population

Patients who were diagnosed with lung cancer between 1 January 2003 and 31 December 2018 were followed-up from their date of registration to the end of 2020, in order to determine their survival rates.

**Figure 1:**
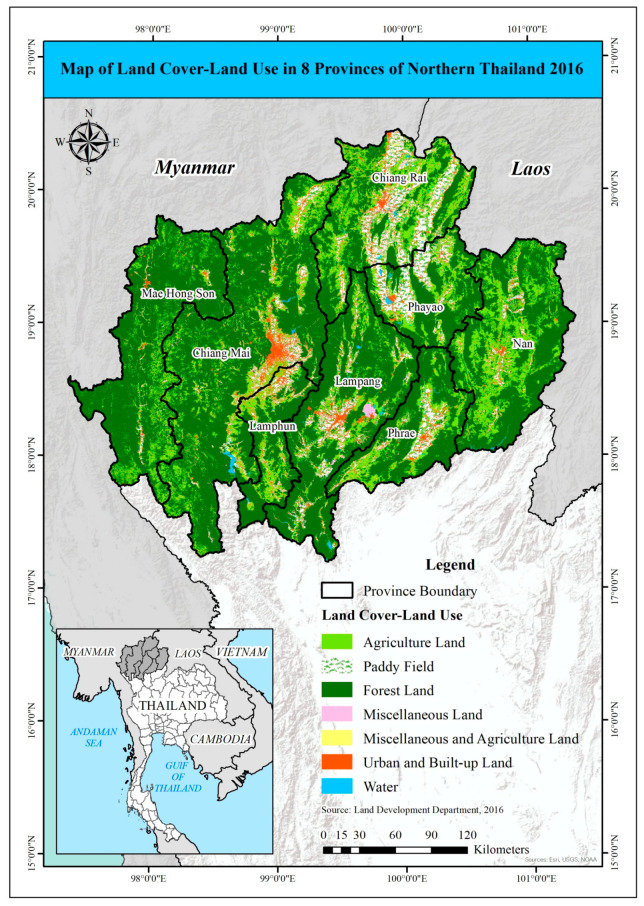
Land use map of 8 provinces in northern Thailand.

This study focused on Thailand’s northern region. This region comprises the provinces of Chiang Rai, Mae-Hong-Son, Chiang Mai, Phayao, Lamphun, Lampang, Phrae, and Nan. The majority of northern Thailand is hilly, and it is the source of several important rivers. The north–south oriented hill ridges run parallel from west to east and are intersected by several major valleys. The northern region’s agricultural land is estimated to be 6,368,630 hectares, accounting for 40% of total land use, as shown in Figure 1. Of this, approximately 41% is paddy fields and 32% is field crops [39].

### 2.2. Exposure Assessment for Time-Updated Variables

We obtained hourly monitoring data for PM2.5, PM10, NO_2_, and O_3_ levels from the Copernicus Atmosphere Monitoring Service (CAMS) of the European Centre for Medium-Range Weather Forecasts (ECMWF) [40,41]. This is the latest global reanalysis dataset of atmospheric composition, consisting of three-dimensional time-consistent atmospheric composition fields that include aerosols and chemical species. The dataset can be used for climatology computations to analyze trends, examine models, compare them to other re-analyses, or serve as the boundary conditions for regional models over time [42]. The annually averaged concentrations of PM2.5 (µg/m^3^), PM10 (µg/m^3^), NO_2_ (ppb) and O_3_ (ppb) were then calculated based on the hourly concentration in each district of upper northern Thailand. We then linked the annually averaged concentration of each pollutant to the district listed in each patient’s address and the calendar year of their diagnosis obtained from the Chiang Mai Cancer Registry and updated every year until either patient death, patient lost to follow-up or loss of data due to censoring. It was assumed that the patients’ recorded addresses were where they lived and subsequently died.

### 2.3. Baseline and Follow-Up Data

The Chiang Mai Cancer Registry provided individual-level information for each cancer patient at diagnosis, including demographics (gender, age, body mass index (BMI), smoking history, and alcohol-use history) and cancer characteristics (cancer stage—SEER staging: localized, regional, or metastasis). Even though the role of alcohol consumption in the occurrence of lung cancer is still controversial, several studies [43,44,45,46] have reported a strong positive association between drinking alcohol and smoking. There might be confounding effect between these variables. In addition, a previous study among patients with non-small-cell lung cancer in the US also found that those patients with alcohol-abuse habits have worse outcomes than non-alcohol-abusing patients [47]. Alcohol-use history was therefore included in the analysis to allow for consideration of its effect on the mortality of lung cancer patients. Every year, the concentrations of PM2.5, PM10, NO_2_, and O_3_ that each patient was exposed to were measured by using the pollution dataset detailed in Section 2.2.

### 2.4. Statistical Analysis

The baseline characteristics are presented as medians and interquartile ranges (IQRs) for the continuous variables, and as frequencies and percentages for the categorical variables. The follow-up time was calculated from the date of diagnosis to either the date of death, regardless of the cause, to the last follow-up date, or to loss of data due to censoring by using the end of the study period (31 December 2020), depending on which came first.

The overall rate of death, and the rates for each variable, were calculated as the number of deaths divided by the total number of person years of follow-up (PYFU). Confidence intervals (CIs) for the mortality rates were based on a Poisson distribution. Survival rates were created by using Kaplan–Meier curves, and log-rank tests were used to test for significance in the difference between the survival probabilities of the groups for each variable.

Cox proportional hazard models were used to investigate any associations between the risk of death among lung cancer patients and the risk factors, including gender, age, cancer stage, smoking history, alcohol-use history, calendar year of enrollment, and time-updated PM2.5, PM10, NO_2_, and O_3_ concentrations. All of the continuous variables were grouped using quartiles, and considered for dichotomization where appropriate (except for BMI with categories: <18.5 and ≥18.5 kg/m^2^, due to the cut-off point recommended in [48]). Factors associated with the risk of death with *p*-value < 0.25 in the univariate analysis were included in the multivariate analysis via a backward elimination procedure, except for variables with a lot of missing values or high correlations (multicollinearity). All analyses were performed by using STATA (version 12).

### 2.5. Ethical Approval

Ethical approval was granted by the Chiang Mai University Ethics Committee (No 200/2021) in the Faculty of Medicine.

## 3. Results

A total of 9820 lung cancer patients were registered between January 2003 and December 2018, 5892 (60%) of whom were males. For the baseline, the median age was 64.0 years (IQR: 56.0–71.7) and the median BMI was 20.1 kg/m^2^ (IQR: 17.8–22.7). For the residential concentration of air pollutants at diagnosis, the median for PM2.5 was 37.4 µg/m^3^ (IQR: 33.8–41.1), the median for PM10 was 52.1 µg/m^3^ (IQR: 47.1–57.1), the median for NO_2_ was 7.6 ppb (IQR: 5.6–8.8) and the median for O_3_ was 36.2 ppb (IQR: 34.9–37.3). For the lung cancer staging, patients were divided into three groups: 21% for localized, 19% for regional, and 61% for metastatic. Furthermore, 78% of patients had a history of smoking, while 55% had a history of alcohol use. The median duration of follow-up was 1.0 years (IQR: 0.52–3.52). During the follow-up period, the median survival time was 0.52 years (IQR: 0.19–1.27).

### 3.1. Baseline Characteristics and Mortality Rate

A total number of 9170 patients died from all causes, with 13,451 PYFU, giving an overall mortality rate of 68.2 per 100 PYFU (95% CI: 66.8–69.6) (Table 1). The mortality rate was 71.8 per 100 PYFU in men (95% CI: 70.7–73.7) and 63.6 per 100 PYFU in women (95% CI: 61.3–65.4). Age at diagnosis ≥ 60 years revealed a high mortality rate of 77.6 per 100 PYFU (95% CI: 75.6–79.6). Being underweight with low BMI had high mortality rates of, specifically, 75.2 per 100 PYFU for weight <50 kg (95% CI: 72.7–77.9), and 77.8 per 100 PYFU for BMI <18.5 kg/m2 (95% CI: 73.8–81.9). Concerning the three cancer stages, the highest mortality rate was found for the metastatic stage (108.0 per 100 PYFU; 95% CI: 105.2–110.9). Smoking and alcohol-use history also provided high mortality rates of 73.6 per 100 PYFU (95% CI: 71.8–75.6) and 69.5 per 100 PYFU (95% CI: 67.2–71.9), respectively. Finally, there was only a small difference for the calendar year of enrollment.

### 3.2. Risk Factors Associated with Death

The results of the uni- and multi-variate analyses for determining the risk factors for death in the lung cancer patients are reported in Table 2. In the univariate analysis, being male, older age, lower BMI, cancer in the metastatic stage, a history of smoking or alcohol-use, enrollment between 2003 and 2010, and time-updated residential concentrations of PM2.5 and PM10, were all associated with a higher risk of death in the lung cancer patients (all *p*-values ≤ 0.001), but as the time-updated residential concentrations of NO_2_ and O_3_ showed *p*-values of 0.543 and 0.782, respectively, the residential concentrations of NO_2_ and O_3_ were not included in the multivariate model. However, we found that BMI, smoking history, and alcohol-use history had a lot of missing values (53.4%, 20.3%, and 33.5%, respectively), so including these variables would have excluded a large number of patients from the multivariate analysis and could have led to invalid results. Therefore, in the multivariate analysis, those variables with a lot of missing values were excluded. In addition, the residential concentration of PM10 was also excluded due to its correlation with the residential concentration of PM2.5. Thus, the multivariate analysis included only gender, age, cancer stage, calendar year of enrollment, and time-updated residential concentration of PM2.5. We found that all included parameters were independently associated with the risk of death (all *p*-values < 0.001). Specifically, the metastatic stage was associated with a higher risk of death with the highest adjusted hazard ratio (aHR) = 2.13 (95% CI: 2.01–2.25). Meanwhile, being male (aHR = 1.17; 95% CI: 1.11–1.22), being older (aHR = 1.28; 95% CI: 1.22–1.33), the regional cancer stage (aHR = 1.32; 95% CI: 1.23–1.41), enrolling before 2010 (aHR = 1.30; 95% CI: 1.24–1.36), and time-updated residential concentration of PM2.5 (aHR = 1.06; 95% CI:1.01–1.11) were also associated with a higher risk of death.

### 3.3. Survival Probabilities

**Figure 2:**
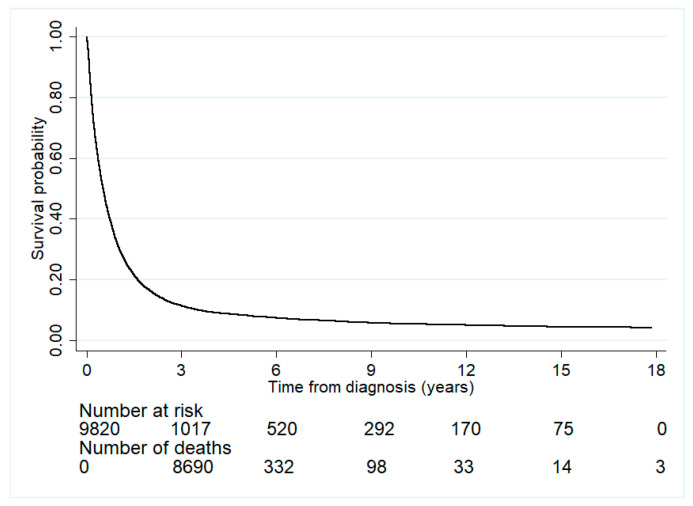
The survival rates of the lung cancer patients. Number at risk represents the number of patients who survived at each time point from diagnosis. Number of deaths represents the number of patients who died during the period between a previous time point to a specific time point.

**Figure 3:**
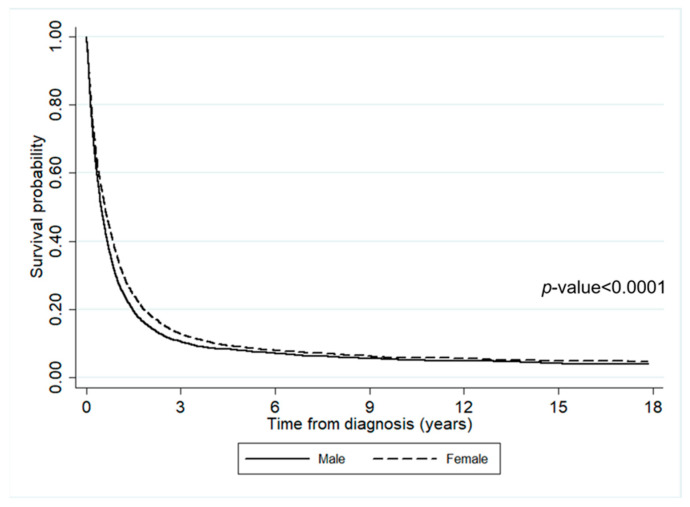
The survival rates of lung cancer patients according to gender.

The impact of diagnosis time on survival is illustrated in Figure 2. Within the first three years of diagnosis, the survival probability dramatically dropped to 10%, with the number of deaths being 8690. After three years since diagnosis, the survival probability slowly decreased throughout the follow-up period. Only a few people were still alive six years after diagnosis. Additionally, Figure 3 shows the impact of gender on survival time. It can be seen that the survival probability of males was slightly lower than females. 

**Figure 4:**
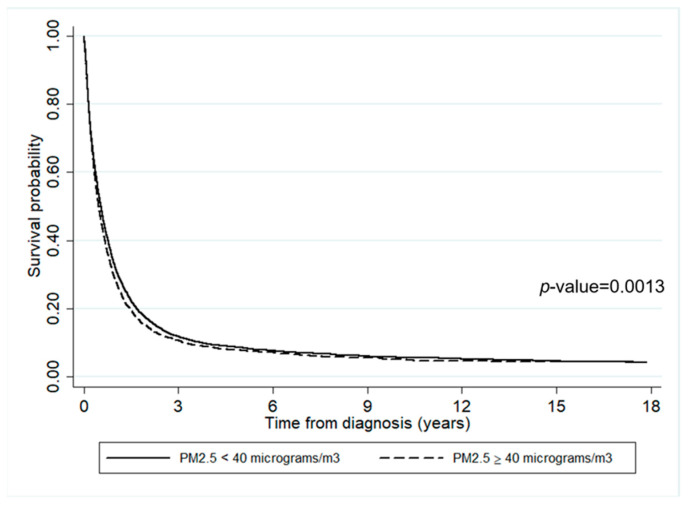
The survival rates of the lung cancer patients according to the annually averaged PM2.5 concentration.

**Figure 5:**
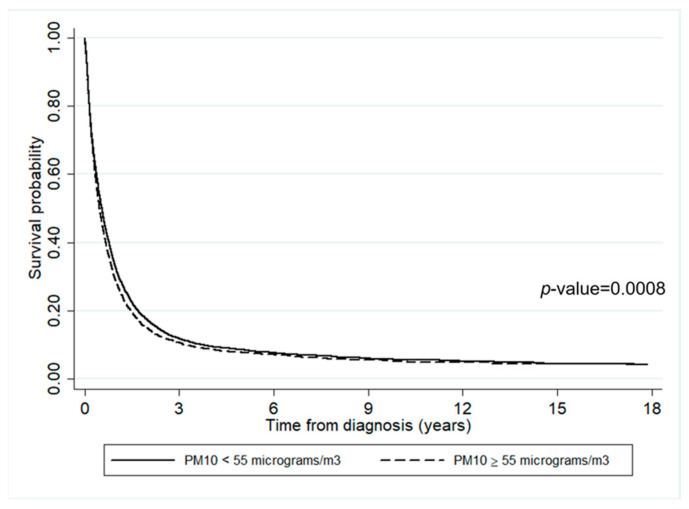
The survival rates of lung cancer patients according to the annually averaged PM10 concentration.

The impact of air pollutants on survival time is presented in Figure 4 and Figure 5. It can be inferred that the survival probabilities of patients who lived in an area where annually averaged PM2.5 ≥ 40 µg/m^3^ was slightly lower than where it was <40 µg/m^3^ (*p*-value = 0.0013). Similarly, the survival probability of those who lived in an area where annually averaged PM10 ≥ 55 µg/m^3^ was slightly lower than where it was <55 µg/m^3^.

## 4. Discussion

We investigated the mortality rate in a large cohort of lung cancer patients in the upper northern area of Thailand. Being male (60%) and being relatively older (median age of 64.0, IOR = 56.0–71.7) displayed higher mortality rates than other factors, which is similar to the findings from other studies [49,50]. This may explain why the overall mortality rate in our study was 68.2 per 100 PYFU, a rate that is consistent with that from the Mazandaran University of Medical Science study on lung cancer patients collected from Tooba Clinic in Sari, Mazandaran Province, Iran (46.8 per 100 person years) [50], but much higher than that reported by the Taiwan Cancer Registry study with/without Chinese herbal treatment (40.24/49.56 per 100 person years) [51]. Note that the contributions of older age and stage of cancer to the risk of death are well-known to be major [52], which was confirmed by the results of the present study.

Most of the risk factors for lung cancer (male, older age, lower BMI, metastatic cancer stage, smoking history, alcohol-use history, enrollment between 2003 and 2010, and time-updated residential concentration of PM) in the univariate analyses are well-known, and our results are consistent with those from other studies [8,53,54,55]. On the other hand, the time-updated residential concentration of NO_2_ and O_3_ are not significantly associated with death from lung cancer, which is different from most other studies [22,23,28], but similar to [56] for O_3_. In the multivariate analyses, we found that all of the input risk factors (male, older age, stage of cancer, early enrollment time, and residential concentration of PM2.5) are associated with a higher risk of death in lung cancer patients, and this is in agreement with the findings from other studies [8,20,22,23,57,58,59].

According to the survival rates, less than 10% of lung cancer patients survived longer than three years after diagnosis, regardless of the residential concentration of air pollution. This result is consistent with the report from the Office for National Statistics that the percentage of lung cancer patients decreased after five years since diagnosis, and only 7.6% of men and 11.3% of women are expected to survive the disease for more than ten years [60]. However, there is a significant difference between the survival times of lung cancer patients living in districts with annually averaged concentrations of PM2.5 < 40 µg/m^3^ and ≥40 µg/m^3^. Similarly, there is a significant difference between those living in areas with annually averaged concentrations of PM10 < 55 µg/m^3^ and ≥55 µg/m^3^. In both cases, the higher the concentration of air pollution, the lower the survival rate. Of note, early enrollment time, which was associated with a higher risk of death, was seen in patients with more exposure to PM2.5 and PM10 pollutants. Therefore, patients living in the areas severely affected by high levels of air pollution had a shorter survival time, which is consistent with the results reported by [20].

Note that lung cancer patients comprise a sensitive group that lives in areas with PM2.5 and PM10 concentrations over both the Thai (25 and 50 µg/m^3^, respectively) and WHO (10 and 20 µg/m^3^, respectively) maximum average allowable concentrations [61]. However, we could not perform our analysis at these levels since the PM data are left-skewed. In other words, the PM2.5 and PM10 in our study areas were higher than the standard levels. Thus, in our analysis, we grouped them using quartiles and by choosing a suitable dichotomization. Together with studies on the impact of air pollution on other diseases [55,62], our results on the effects of PM2.5 and PM10 on lung cancer patients indicate that the severe issue of air pollution, especially in the northern areas of Thailand, should be resolved.

Apart from the air pollution, the causes of the poor survival outcome of patients in this study might be from the quality of the diagnosis and treatment of lung cancer, together with limitations in healthcare resources, including facilities and medical personnel. A computed tomography (CT) scan is routinely performed for diagnosis in Thailand, according to national guideline. However, the image quality is not good enough in some rural hospitals. Recently, the Thoracic Society of Thailand under Royal Patronage developed clinical practice guidelines for lung cancer diagnosis [30]. The evolving technologies for treatment, including surgery and radiotherapy, have rapidly progressed in Thailand in the past ten years [30]. Nevertheless, the accessibility of novel chemotherapeutic or targeted drugs used for advanced stages of lung cancer is limited for most Thai patients [30].

Our study had several strengths. First, this was a very large hospital-based cohort study that was sufficient for providing results on lung cancer mortality and survival rates. Second, the lung cancer patients’ data, as well as the data on air pollutants, were collected over a 15-year period (2003–2020). Finally, this is the first investigation that has applied time-varying covariate analysis to PM data to test whether fine PM, along with other risk factors, affects the survival rate of lung cancer patients. Meanwhile, meteorological factors (such as temperature, humidity, and wind speed) that are significantly associated with lung cancer mortality [22,23] might be considered in a future study.

Nevertheless, the study also has the following limitations. First, the residential concentrations of air pollutants were calculated under the assumption that the patients mostly lived and died in the district in which they were registered. In fact, it is possible that the patients indeed stayed in other districts with higher or lower residential concentrations of PM than their home districts. To resolve this issue in a similar study in the future, we might have to recheck whether the patients mostly lived and died in their registered districts or not, and hence we would have to exclude any patient who does not meet this criterion. Second, many values for weight, BMI, smoking history, and alcohol-use history were missing, and so these factors could not be included in the multivariate Cox proportional hazard analysis. Hence, an appropriate method to impute these missing values, such as the Multivariate Imputation by Chained Equation (MICE) could be applied [63]. This could compensate for the missing values of those variables and lead to adjustment of the multivariate analysis for the mortality risk of lung cancer patients. Another limitation in this study is that the details of treatment for each lung cancer patient recorded in the hospital medical record were not systematically combined with the Chiang Mai Cancer Registry. Therefore, we could not perform any investigation on this aspect. In the case that the treatment data from the hospital medical records and the data in the Chiang Mai Cancer Registry can be systematically combined, analysis on the impact of treatments associated with lung cancer mortality might be performed.

Lastly, the Chiang Mai Cancer Registry has recorded the lung cancer patients’ data based on the IARC CanReg5 tool. The IARC CanReg5 tool does not record the patients’ lung cancer stages nor the type of lung cancer (small cell or non-small cell). Therefore, we were unable to present and analyze these characteristics in this study. As these characteristics can be obtained from the clinical cancer registry, which has not been applied to the Chiang Mai Cancer Registry, in the future, analyses from the clinical cancer registry could provide a better understanding of the association between lung cancer mortality and risk factors.

## 5. Conclusions

In summary, we found that the mortality rate of lung cancer patients in upper northern Thailand was high, and the mortality risk factors were sex (male), older age, the stage of cancer, and the amount and period of exposure to PM2.5 and PM10. The fact that concentrations of these air pollutants comprise one of the risk factors associated with a higher risk of death from lung cancer indicates that air pollution is a major problem in the upper northern part of Thailand that needs to be addressed. While the severe problem of PM2.5 and PM10 is still waiting to be fixed, lung cancer patients who live in such areas should reduce their exposure to fine particulate matter in order to increase their survival rate. 

## Figures and Tables

**Table 1:** Baseline characteristics of the study population and mortality rate.

Characteristic	Survived (*n* (%))	Died (*n* (%))	PYFU	Mortality Rate *	95% CI
Overall	650 (7%)	9170 (93%)	13,451	68.2	66.8–69.6
Gender					
Male	358 (6%)	5534 (94%)	7705	71.8	70.0–73.7
Female	292 (7%)	3636 (93%)	5746	63.3	61.3–65.4
Age at diagnosis (years)					
<60 years	295 (8%)	3355 (92%)	5953	56.4	54.5–58.3
>=60 years	355 (6%)	5815 (94%)	7498	77.6	75.6–79.6
BMI (kg/m^2^)/5243					
<18.5 kg/m^2^	73 (5%)	1401 (95%)	1802	77.8	73.8–81.9
>=18.5 kg/m^2^	379 (12%)	2724 (88%)	5833	46.7	45.0–48.5
Cancer stage/257					
Localized	288 (15%)	1681 (85%)	4893	34.4	32.7–36.0
Regional	152 (8%)	1645 (92%)	2877	57.2	54.5–60.0
Metastasized	174 (3%)	5623 (97%)	5205	108.0	105.2–110.9
Smoking history/1990					
Yes	349 (6%)	5788 (94%)	7859	73.6	71.8–75.6
No	198 (12%)	1495 (88%)	2988	50.0	47.6–52.6
Alcohol-use history/3285					
Yes	238 (7%)	3346 (93%)	4817	69.5	67.2–71.9
No	258 (9%)	2693 (91%)	4331	62.2	59.9–64.6
Calendar year of enrollment					
2003–2010	205 (3%)	4917 (97%)	7413	66.3	64.5–68.2
2011–2018	445 (9%)	4253 (91%)	6038	70.4	68.3–72.6

* per 100 PYFU (person years of follow-up). CI, confidence interval; BMI, body mass index.

**Table 2:** Risk factors associated with death among the lung cancer patients.

Characteristic	Univariate Analysis					Multivariate Analysis		
	Deaths	Total	HR	95%CI	*p*-Value *	aHR	95%CI	*p*-Value *
**At diagnosis**								
Male	5534	5892	1.13	1.08–1.18	<0.001	1.17	1.11–1.22	<0.001
Age ≥ 60 years	5815	6170	1.21	1.16–1.26	<0.001	1.28	1.22–1.33	<0.001
BMI < 18.5 kg/m^2^	1401	1474	1.48	1.39–1.58	<0.001	-	-	-
Regional cancer stage	1645	1797	1.25	1.17–1.34	-	1.32	1.23–1.41	-
Metastatic cancer stage	5623	5797	1.97	1.86–2.08	<0.001	2.13	2.01–2.25	<0.001
Smoking history	5788	6137	1.39	1.32–1.48	<0.001	-	-	-
Alcohol-use history	3346	3584	1.12	1.06–1.18	<0.001	-	-	-
Enrolled between 2003 and 2010	4917	5122	1.19	1.14–1.24	<0.001	1.30	1.24–1.36	<0.001
**Time-updated variables**								
Residential concentration of PM2.5 ≥ 40 (µg/m^3^)	-	-	1.08	1.03–1.13	0.001	1.06	1.01–1.11	0.018
Residential concentration of PM10 ≥ 55 (µg/m^3^)	-	-	1.08	1.03–1.13	0.001	-	-	-
Residential concentration of NO_2_ ≥ 8.7 ppb	-	-	1.01	0.97–1.06	0.543	-	-	-
Residential concentration of O_3_ ≥ 37.8 ppb	-	-	0.99	0.95–1.04	0.782	-	-	-

* *p*-value from partial likelihood ratio test. HR, hazard ratio; aHR, adjusted hazard ratio.

## Data Availability

The datasets used and/or analyzed during the current study are available from the corresponding author on reasonable request.
